# A *Drosophila* model targets Eiger/TNFα to alleviate obesity-related insulin resistance and macrophage infiltration

**DOI:** 10.1242/dmm.050388

**Published:** 2023-11-06

**Authors:** Zhasmine Mirzoyan, Alice Valenza, Sheri Zola, Carola Bonfanti, Lorenzo Arnaboldi, Nicholas Ferrari, John Pollard, Valeria Lupi, Matteo Cassinelli, Matteo Frattaroli, Mehtap Sahin, Maria Enrica Pasini, Paola Bellosta

**Affiliations:** ^1^ Department of Computational, Cellular and Integrative Biology (CIBIO), University of Trento, 38123 Trento, Italy; ^2^ Department of Biosciences, University of Milan, 20133 Milan, Italy; ^3^ Department of Biology, University of Ankara, 06110 Ankara, Turkey; ^4^ Department of Pharmacology, University of Milan, 20133 Milan, Italy; ^5^ Department of Medicine, NYU Langone Medical Center, 10016 New York, USA

**Keywords:** *Drosophila* model, Adipose chronic inflammation, Eiger/TNFα signaling, Insulin resistance, Lipid metabolism, Obesity, Metabolic disorders

## Abstract

Obesity is associated with various metabolic disorders, such as insulin resistance and adipose tissue inflammation (ATM), characterized by macrophage infiltration into adipose cells. This study presents a new *Drosophila* model to investigate the mechanisms underlying these obesity-related pathologies. We employed genetic manipulation to reduce ecdysone levels to prolong the larval stage. These animals are hyperphagic and exhibit features resembling obesity in mammals, including increased lipid storage, adipocyte hypertrophy and high circulating glucose levels. Moreover, we observed significant infiltration of immune cells (hemocytes) into the fat bodies, accompanied by insulin resistance. We found that attenuation of Eiger/TNFα signaling reduced ATM and improved insulin sensitivity. Furthermore, using metformin and the antioxidants anthocyanins, we ameliorated both phenotypes. Our data highlight evolutionarily conserved mechanisms allowing the development of *Drosophila* models for discovering therapeutic pathways in adipose tissue immune cell infiltration and insulin resistance. Our model can also provide a platform to perform genetic screens or test the efficacy of therapeutic interventions for diseases such as obesity, type 2 diabetes and non-alcoholic fatty liver disease.

## Teaser

**Summary:** This study presents a new *Drosophila* model to explore obesity-related concerns such as insulin resistance and adipose tissue inflammation, which are ameliorated by reducing Eiger/TNFα signaling.

## INTRODUCTION

Obesity is a multifactorial disease influenced by genetic, environmental and behavioral factors (Kahn et al., 2019). In obesity, impairment in fat metabolism results in elevated levels of circulating free fatty acids (FFAs) and pro-inflammatory cytokines, which contribute to the development of chronic low-grade tissue inflammation, with macrophages infiltrating various tissue, which in fat is called adipose tissue macrophage infiltration (ATM) (Kawai et al., 2021). Macrophages play an active role in tissue remodeling and repair. In metabolically active tissues such as adipose, they accumulate and become activated in response to soluble factors, releasing pro-inflammatory molecules like cytokines (TNFα) and chemokines. These molecules contribute to sustaining the chronic inflammatory state through mechanisms that are not yet fully understood (Cai et al., 2022; Dahik et al., 2020; Gregor and Hotamisligil, 2011; Kawai et al., 2021). Furthermore, chronic inflammation is associated with increased oxidative stress through the production of high levels of reactive oxygen species (ROS) (Rani et al., 2016), often responsible for JNK/SAPK/p46 signaling activation and apoptosis (Hotamisligil, 2003). Moreover, dysregulation of fat metabolism contributes to the development of insulin resistance, a risk factor for metabolic disorders (Deprince et al., 2020; Yang et al., 2018) and increases susceptibility to cardiovascular disease (Nakamura and Sadoshima, 2020).

In recent years, a defined role in humans for steroid hormones, including estrogen and testosterone, has been associated with their role in controlling the timing and progression of maturation as well as association with metabolic disorders, including obesity. In particular, low levels of estrogen after menopause, or testosterone in hypogonadism, increases the risk of developing obesity or other metabolic complications (Fan et al., 2019; Sebo and Rodeheffer, 2021; Wittert and Grossmann, 2022). Those observations reveal a form of mechanistic regulation that may demonstrate striking similarities across distant species, governing developmental processes in flies and humans (Kannangara et al., 2021).

By taking advantage of the conserved functional relationships between the *Drosophila* immune cells (hemocytes) and the regulation of adipokines in the fat body (FB), a metabolic tissue with physiological functions similar to those of mammalian adipose tissue and liver (Liu et al., 2009), we previously demonstrated that animals with reduced ecdysone have an obese-like phenotype and show high levels of hemocytes infiltrating the FB, mimicking chronic inflammation in obesity (Valenza et al., 2018). This was also accompanied by increased ROS and activation of p46/JNK/SAPK signaling (Valenza et al., 2018).

The *Drosophila* immune response is primarily mediated by the hemocytes (macrophage-like) circulating in the hemolymph that are present at all stages of the life cycle (Buchon et al., 2014; Lemaitre and Hoffmann, 2007; Leulier and Lemaitre, 2008). Hemocytes are essential mediators in the cell–cell communication process: they have been shown to mediate a response between the FB and other organs to control the abnormal proliferation of tumor-like cells (Parisi et al., 2014) and to promote proliferation in response to ROS produced during cell death in the process of regeneration of the imaginal discs (Fogarty et al., 2016).

A complex network of humoral factors coordinate these signals, including Eiger, the fly homolog of TNFα (Droujinine and Perrimon, 2016; Igaki and Miura, 2014). Eiger is secreted by cells of the FB to act on the insulin-producing cells (IPCs) to regulate the nutrient response and in the interaction between metabolism and immune signaling (Agrawal et al., 2016; Colombani and Andersen, 2023; Meschi and Delanoue, 2021). Notably, dysregulation of TNFα signaling has been associated with metabolic disorders such as insulin resistance and obesity in mammals (Igaki and Miura, 2014; Tzanavari et al., 2010; Yaribeygi et al., 2019).

Expanding our earlier discovery (Valenza et al., 2018), which showed that reducing ecdysone levels led to animals with traits resembling obesity-related disorders, we conducted an in-depth analysis of a model called OBL (obese-like larvae). Our investigation found that OBL larvae exhibited hyperphagia and progressively accumulated triglycerides (TGs), FFAs and glucose throughout development. These changes are consistent with what is observed in human obesity, in which excess nutrients are stored as fats and circulating glucose levels are elevated. We found TG accumulation in the cells of the FB, which increased in size, and in the cells of the cardiac tube, which showed the presence of lipid droplets and contraction defects. The OBL animals did not respond to systemic insulin signaling regulation and were hyperglycemic, and cells of the FBs acquired insulin resistance. The OBL animals also showed a significant increase in the production of Eiger/TNFα in the FB and in hemocytes, and attenuation of Eiger signaling reduced the infiltration of hemocytes in the adipose cells, which also regained sensitivity to insulin treatment.

Finally, we administered the anti-diabetic drug metformin and natural antioxidants anthocyanins (ACN) to OBL animals, substantially reducing hemocyte infiltration into the FB. These results further validate the potential of this new model for drug screening against obesity and to gain deeper insights into the regulatory mechanisms of chronic inflammation and insulin resistance in obesity and related metabolic disorders.

## RESULTS

### High-fat diet (HFD) and mutations in *brummer* lead to low-grade hemocyte infiltration in the FB

**Fig. 1.**
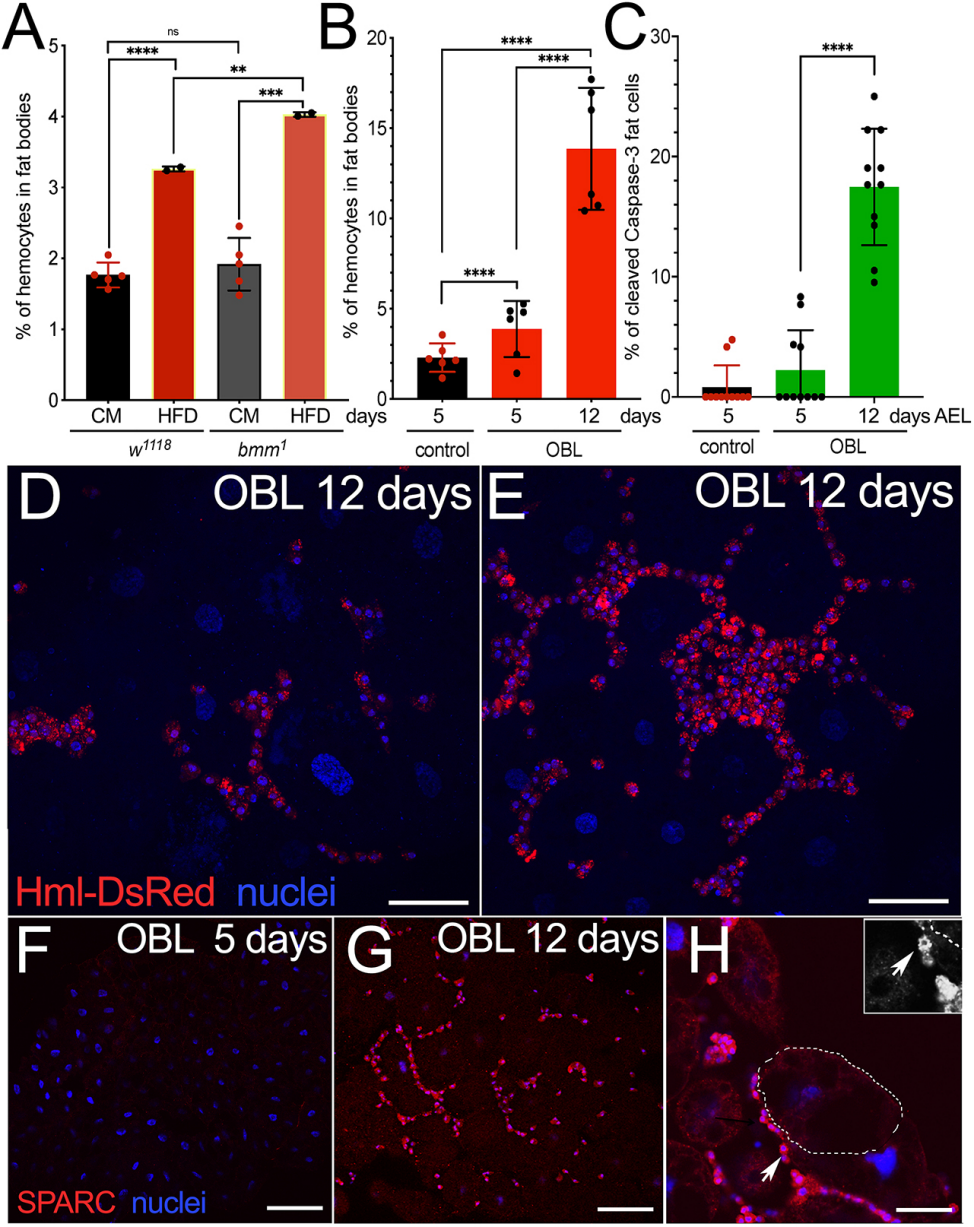
**Hemocyte infiltration in the fat body (FB) of larvae fed a high-fat diet (HFD) or mutants for *brummer* phenocopy low-grade chronic inflammation associated with obesity in humans.** (A) Percentage of hemocytes infiltrating the FB of wild-type (*w^1118^*) animals fed an HFD or corn meal (CM) and in animals mutant for *brummer* (*bmm^1^*) measured at 5 days after egg laying (AEL). (B) Hemocytes in the FB measured at 5 days AEL in control *P0206-w^1118^* and in *P0206-NOC1*, herein called OBL (see Fig. S1), at 5 and 12 days AEL. Quantification in the FB of hemocytes marked by Hml-DsRed expressed as a percentage of DsRed cells from the total number of cells quantified by nuclear staining using Hoechst. (C) Quantification of apoptosis in cells of the FB analyzed using anti-caspase 3 antibody in OBL animals at 12 days AEL. The dots in A and B indicate the number of experiments (at least ten animals were used for each time point and genotype), and data in C represent one of two experiments done, with the dots representing the number of animals used in the experiment. (D,E) Confocal images of FB from OBL animals at 12 days AEL showing the hemocytes marked by Hml-DsRed expression (Makhijani et al., 2011). (F-H) Confocal images of FB from *w^1118^* animals at 5 days (F) and of FB from OBL animals at 12 days AEL (G,H), showing the hemocytes, stained using anti-SPARC antibody [panel G is from Valenza et al. (2018); image reproduced under the terms of the CC-BY 4.0 license]. F highlights the crown-like structure formed by the hemocytes surrounding a fat cell. The inset in H shows hemocytes (arrow) with the typical macrophage-like morphology at higher magnification. Scale bars: 50 µm (D,E,H), 25 µm (F,G). Statistical analysis was performed using unpaired two-tailed Student's *t*-test. Error bars indicate s.d. ns, not significant; **P<0.01, ***P<0.001 and ****P<0.0001.

We previously demonstrated that reducing ecdysone in the prothoracic gland results in hyperphagic animals that accumulate fats and exhibit low-grade hemocyte infiltration in the FB (Valenza et al., 2018), resembling the infiltration of macrophages observed in adipose tissues of obese people (Kawai et al., 2021). In this model, we genetically reduced ecdysone production from the beginning of development by lowering protein synthesis in the prothoracic gland (Destefanis et al., 2022). This led to developmental delay and inhibition of the progression of the animals to the pupal stage. Consequently, the animals continued growing and exhibited FB tissue enlargement, a condition that persisted until their death at ∼20 days after egg laying (AEL) (Fig. S1) (Valenza et al., 2018). To analyze whether the infiltration of hemocytes was dependent on changes in lipid metabolism, we fed *w^1118^* larvae an HFD (Diop et al., 2017), or analyzed whether a mutation in the lipase *brummer*, the ortholog of human *ATGL* (*PNPLA2*) (Gronke et al., 2005) could influence hemocyte migration. For easy *in vivo* visualization of hemocytes, we introduced the *Hml-DsRed* construct under the *Hemolectin* promoter (Makhijani et al., 2011) into the OBL line and the control, and analyzed the expression of the DsRed fluorescent protein in their FB. These data showed that feeding animals an HFD significantly induced the migration of hemocytes in the FB of *w^1118^* larvae, visible at 5 days AEL (Fig. 1A). In addition, this effect was further enhanced in animals mutant for *brummer*, suggesting that dysregulated lipid signaling, or lipid accumulation, favored the migration of hemocytes in the FB. Using the same *Hml-DsRed* reporter, we then quantified the hemocytes present in the fat of the OBL animals and observed a significant number of hemocytes infiltrating the FB of larvae at 5 days AEL, which further increased at 12 days AEL (Fig. 1B). Furthermore, their presence was accompanied by a significant level of apoptosis in the FB (Fig. 1C). Analysis in other larval tissues showed that hemocytes preferentially infiltrate the FB, as few *Hml-DsRed* cells were detected in the brain, gut or other tissues (Fig. S2). A closer look at the cellular imaging of the *Hml-DsRed* cell distribution in the FB of the OBL animals at 12 days AEL revealed that hemocytes form the typical ‘crown’ structures around the fat cells (Fig. 1D,E), a characteristic also observed in the adipose tissues of obese people with ATM (Kawai et al., 2021). We previously observed similar structures using antibodies against Secreted protein, acidic, cysteine-rich (SPARC) (Martinek et al., 2002), expressed in basal lamina and in the membranes, to visualize the hemocyte membranes at the border with the FB (Fig. 1F,G) (Valenza et al., 2018). Indeed, a picture at higher magnification of *Drosophila* hemocytes shows the typical jagged morphology of the membranes (Fig. 1H) described also in human macrophages. In summary, just like larvae fed an HFD or larvae with genetic alterations of their lipid metabolism (*brummer* mutation), our OBL animals exhibit the migration of immune cell hemocytes (macrophages) into the adipose tissue, a condition also described in human obesity.

### OBL animals exhibit elevated levels of TGs, FFAs and monounsaturated fatty acids (MUFAs) in the whole body, accompanied by lipid accumulation and contractility defects in cardiac cells

**Fig. 2.**
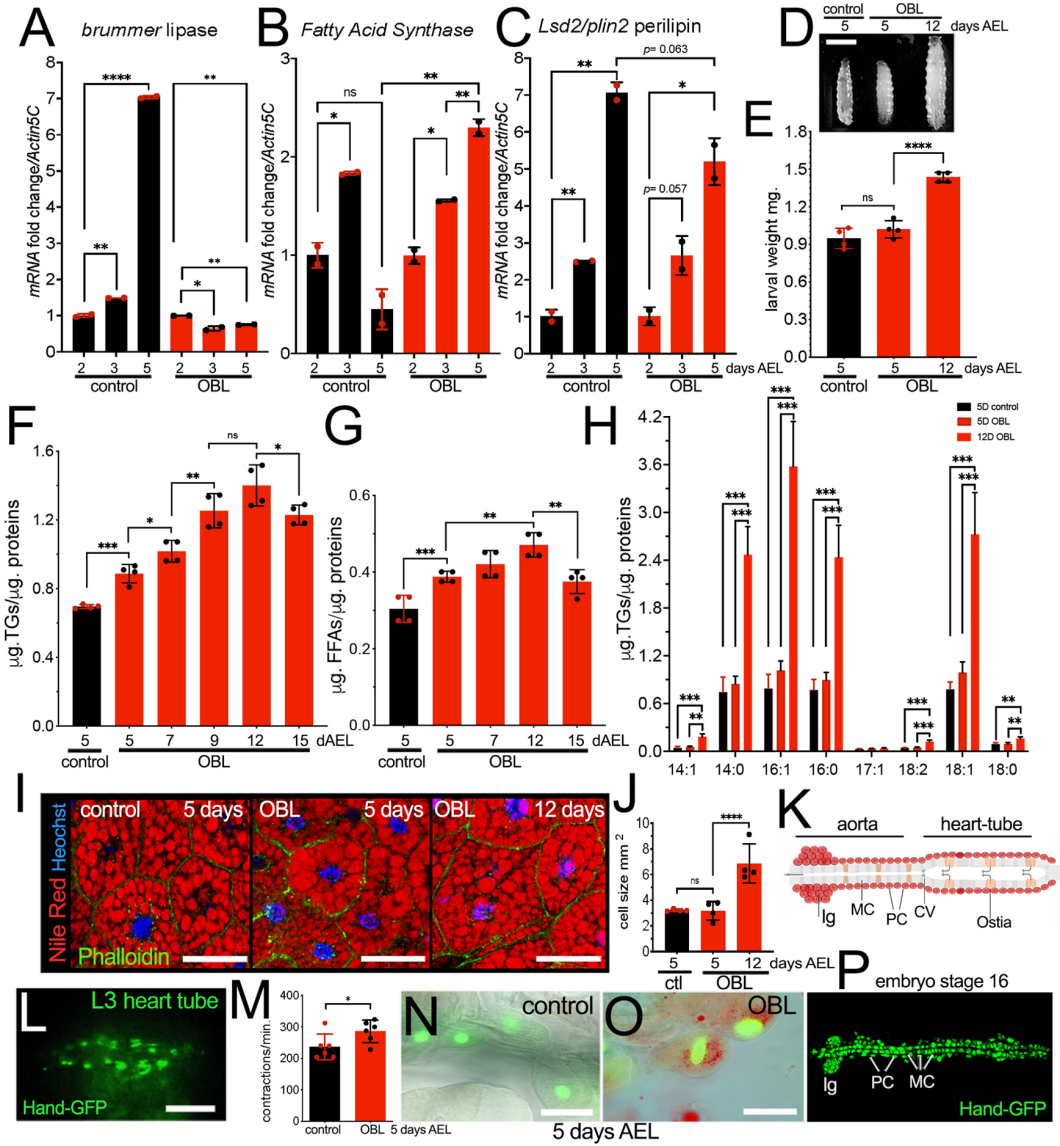
**OBL animals increase fatty acid synthesis and accumulate triglycerides (TGs) and free fatty acids (FFAs), accumulating lipids in FB and pericardial cells.** (A-C) qRT-PCT showing the level of *brummer*, *Fatty acid synthase*, and *Plin2* mRNA in whole larvae of the indicated genotype collected 2, 3 and 5 days AEL. (D,E) Photographs of control *w^1118^* or OBL larvae at 5 or 12 days AEL (D) and relative weight (E). (F) TG content in whole larvae at the indicated time (days AEL). (G) FFAs from whole larvae collected at the indicated time (days AEL). (H) Lipidomic analysis of fats from whole larvae of the indicated genotype at 2, 5 and 12 days AEL. (I) Confocal images of cells from the FB of larvae of the indicated genotype; fat is stained with Nile Red and nuclei with Hoechst. (J) Size analysis of the cells from the FB of animals of the indicated genotype; analysis was performed using ImageJ from confocal images. (K) Schematic of the heart tube (cardiac tube) in embryos, showing the relative cell types*.* The heart tube contains openings called muscular ostia (Ostia) surrounded by muscle cells; the ostia function as valves to allow the entry of hemolymph into the heart tube. As the heart tube contracts, the hemolymph is propelled forward, filtered and pushed out of the heart tube into the aorta, the main artery of the circulatory system in flies. The cardiovascular valve (CV) ensures the unidirectional hemolymph flow from the heart tube to the aorta, preventing backflow. Myocardial cells (MC) and pericardial cells (PC) are shown, and expression is also detected in the lymph glands (lg). Anterior is to the left. (L) Photograph of the cardiac tube of a third-instar (L3) larva; Hand-GFP expression visualizes the cardiac cells. (M) Analysis of cardiac contraction in larvae of the indicated genotype at 5 days AEL. Dots in the graphs show the number of experiments performed. (N,O) Higher magnifications of the cardiac cells from third-instar larvae in M; cells are marked by the expression of Hand-GFP and stained for lipid contents using Nile Red. Scale bars: 1 mm (D), 100 µm (L), 50 µm (I) and 20 µm (N,O). Statistical analysis was performed using unpaired two-tailed Student's *t*-test, except for data in H, for which *P-*values were calculated using one-way ANOVA with Tukey multiple comparisons. Error bars indicate s.d. ns, not significant; *P<0.05, **P<0.01, ***P<0.001 and ****P<0.0001.

Transcriptional analysis of key enzymes that regulate fat metabolism showed a decrease in *brummer* mRNA expression, but increase in *Fatty acid synthase* (*FAS*; *FASN1*) and *Perilipin* (*Lsd-2*) mRNA expression, in the OBL larvae at early larval development (Fig. 2A-C). The OBL animals were hyperphagic and increased in size and weight over time (Fig. 2D,E). Analysis of whole-body TGs showed that the OBL larvae accumulate TG (Fig. 2F) and increase their levels of FFAs (Fig. 2G). Lipidomic analysis (Fig. 2H) confirmed the increase in *de novo* synthesis and accumulation of TGs, with a significant increase in saturated fatty acids (SFAs) (C14:0 myristic C16:0 palmitic) over the total lipids and MUFAs (C16:1 and C18:1) in OBL animals (Fig. S3). Notably, in humans, SFAs (C14:0 and C16:0) are associated with the activation of macrophages and the development of inflammation in metabolic pathologies such as obesity and type 2 diabetes (Sarabhai et al., 2020; Silva Figueiredo et al., 2017; Wrzosek et al., 2022). OBL larvae also showed a significant increase in lipids in the cells of the FB, visualized by Nile Red staining (Fig. 2I), which paralleled the increase in the size of the cells (Fig. 2J).

Obesity is associated with cardiac dysfunction. In flies, cardiac cells develop early in the embryo and are already contracting at stage 16, when the cardioblasts, which form the cardiac tube, differentiate into contractile cardiomyocytes or myocardial cells (MCs), while the pericardial cells (PCs) surround the MCs to filter the hemolymph (Fig. 2K) (Souidi and Jagla, 2021). We expressed the reporter Hand-GFP to visualize the cardiac cells (Fig. 2P), and, using a video tracking system, we analyzed the frequency of Hand-GFP contraction in the two genotypes (Fig. 2L shows Hand-GFP expression in larvae 5 days AEL). These data show increased contraction frequency in cells in OBL larvae as early as 5 days AEL (Fig. 2M). Furthermore, these cells showed an accumulation of lipid droplets (Fig. 2O) compared to the control (Fig. 2N), suggesting that lipid dysregulation in OBL larvae mirrors the lipid accumulation seen in cardiomyopathies induced by HFD and high-sugar diet (HSD) (Birse et al., 2010; Na et al., 2013).

### OBL animals exhibit impaired systemic insulin regulation and insulin resistance in FB cells

**Fig. 3.**
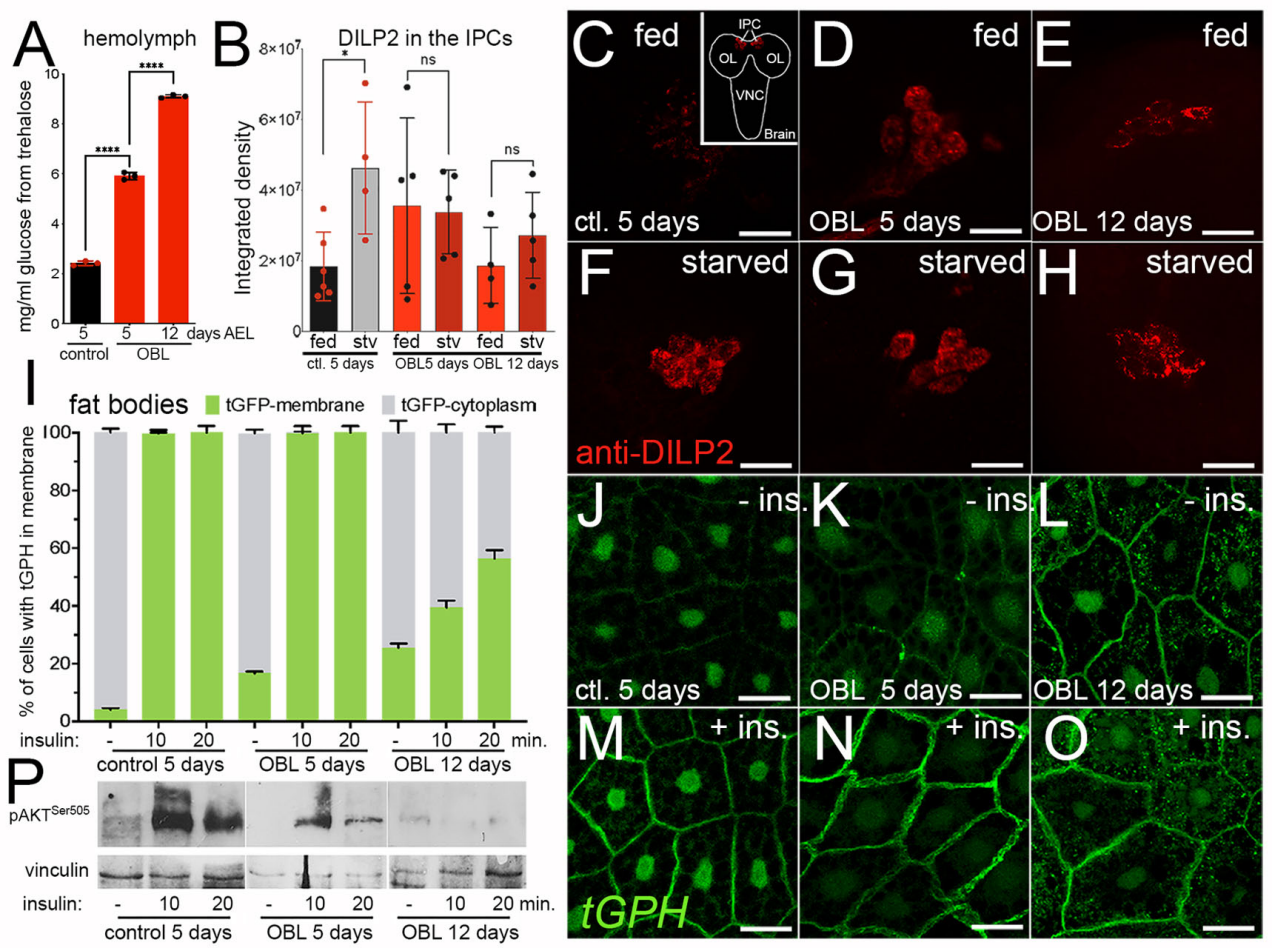
**OBL larvae are hyperglycemic, have impaired systemic insulin signaling and exhibit insulin resistance in the FB.** (A) Hemolymph glucose analysis from animals of the indicated genotypes. At least ten larvae were used for each genotype. (B) DILP2 expression was measured by immunofluorescence using anti-DILP2 antibody in the insulin-producing cells (IPCs) from third-instar larvae raised in corn meal food (fed) or starved for 24 h in PBS (stv). The integrated density of fluorescence was quantified using confocal images as described previously (Parisi et al., 2013). *P*-values were calculated using unpaired two-tailed Student's *t*-test from *n*=10 larvae for each time point and genotype and from at least four independent experiments. Error bars indicate s.d. ns, not significant; *P<0.05, and ****P<0.0001. (C-H) Confocal images of brains from larvae of the indicated age and genotypes raised in normal food (C-E) or kept overnight in PBS (F-H), showing DILP2 expression (red) in the IPCs. The inset in C shows a drawing of the larval brain indicating the IPC position. OL, optic lobe; VNC, ventral nerve cord. Scale bars: 50 µm. (I) Quantification of tGPH-GFP expression in the membrane of the fat cells from control and OBL larvae; the FBs were incubated *ex vivo* with 1 mM insulin for the indicated time. (J-O) Confocal images of FBs expressing the tGPH-GFP reporter from larvae in I, untreated (J-L) treated with insulin for 10 min (M-O). Scale bars: 50 µm. (P) Western blot of lysates from FBs of animals of the indicated age and genotype upon treatment of insulin, showing the level of phosphorylation of Akt at Ser505. Vinculin was used as a control for loading.

Hyperglycemia is a hallmark of metabolic dysfunctions described in flies subjected to diets enriched in sugars (HSD) and fats (HFD) (Birse et al., 2010; Morris et al., 2012; Musselman et al., 2011; Skorupa et al., 2008) that can also lead to insulin resistance in adipocytes (Tabak et al., 2012). OBL larvae are already hyperglycemic at 5 days AEL, as shown by the increased level of glucose (measured as trehalose) in their hemolymph (Fig. 3A). This condition results in an increase in glucose and storage of glycogen in the whole body (Fig. S4). Elevated glucose in the hemolymph has been associated with defects in the activity of *Drosophila* insulin-like peptides (DILPs), in particular of DILP2 (Ilp2) and DILP5 (Ilp5) (Semaniuk et al., 2018; Zhang et al., 2009). DILP2/5 stimulate insulin signaling to control the growth of the animal. They are produced and accumulate in the IPCs to be released on demand by signals from the FB, which senses the levels of nutrients in the hemolymph (Geminard et al., 2009; Meschi and Delanoue, 2021). Analysis of DILP2 localization in the IPCs shows that control animals fed regular food exhibit undetectable levels of DILP2 during feeding conditions (Fig. 3B,C), and, as expected, the level of DILP2 increased in the IPCs upon starvation (Fig. 3B,F). By contrast, in the IPCs of the OBL larvae, DILP2 levels were found abnormally elevated in animals fed regular food, both at 5 and 12 days AEL (Fig. 3D,E), and this amount did not significantly change upon starvation (Fig. 3G,H). These data indicate that the FB from the OBL animals has lost the signals to sense the nutrients and the control over DILP2 homeostasis.

High levels of circulating lipids and reduced glucose uptake indicate insulin resistance. Our findings indicate impaired insulin signaling in OBL larvae. Consequently, we analyzed whether cells within the FB, the primary metabolic organ, exhibit reduced responsiveness to insulin stimulation. In *ex vivo* experiments, we isolated the FBs from control and OBL larvae at 5 or 12 days AEL and incubated them with Schneider’s medium (Thermo Fisher Scientific) containing 1 mM insulin. Activation of insulin signaling was analyzed using the reporter line PH-GFP (tGPH) carrying the pleckstrin homology (PH) domain fused with GFP that translocates to the plasma membrane upon PI3K activation (Britton et al., 2002). These data showed that treating FBs with insulin for 10 min completely induced the translocation of the tGPH reporter into the membrane in control and OBL animals at 5 days AEL (Fig. 3I-K,M,N), whereas in FBs from OBL animals at 12 days AEL only 40% of the tGPH reporter was translocated in the membrane, and this signal only partially increased after 20 min of insulin treatment (Fig. 3I,L,O). Furthermore, we observed a low background of tGPH expression at the membrane in the FB of OBL animals at 12 days AEL, even before adding insulin (Fig. 3L). We also noted tGFP nuclear staining in both fed and starved tissues in these experiments. We attributed this staining to background signals, as previously reported in the original paper by Britton et al. (2002). Consequently, we did not incorporate this staining into our analysis. Next, we analyzed the response of FB cells to insulin stimulation by measuring the level of Akt phosphorylation at Ser505 in lysates of FBs from control and OBL animals collected at 5 and 12 days AEL. These data show that insulin treatment in fats from control animals substantially activates Akt phosphorylation, with a robust signal still present 20 min after treatment (Fig. 3P, left). By contrast, in lysates from OBL larvae, Akt phosphorylation at Ser505 was significantly lower than in control at 5 days AEL and almost undetectable at 12 days AEL (Fig. 3P, middle and right), suggesting that the FB of OBL animals has an impaired response to insulin stimulation and exhibits insulin resistance. Additionally, when examining endogenous insulin signaling levels in the FB using transcription markers like Foxo and 4EBP, which are known targets of InR (Puig et al., 2003; Teleman et al., 2008), it became evident that *4EBP* mRNA expression is significantly reduced in OBL animals compared to that in control at 5 days AEL. Furthermore, this reduction is even more pronounced in OBL animals at 12 days AEL (Fig. S5), strongly suggesting that insulin signaling is compromised in the FB of OBL animals.

### Eiger/TNFα signaling contributes to ROS production, chronic inflammation and insulin resistance in the FB of obese animals

**Fig. 4.**
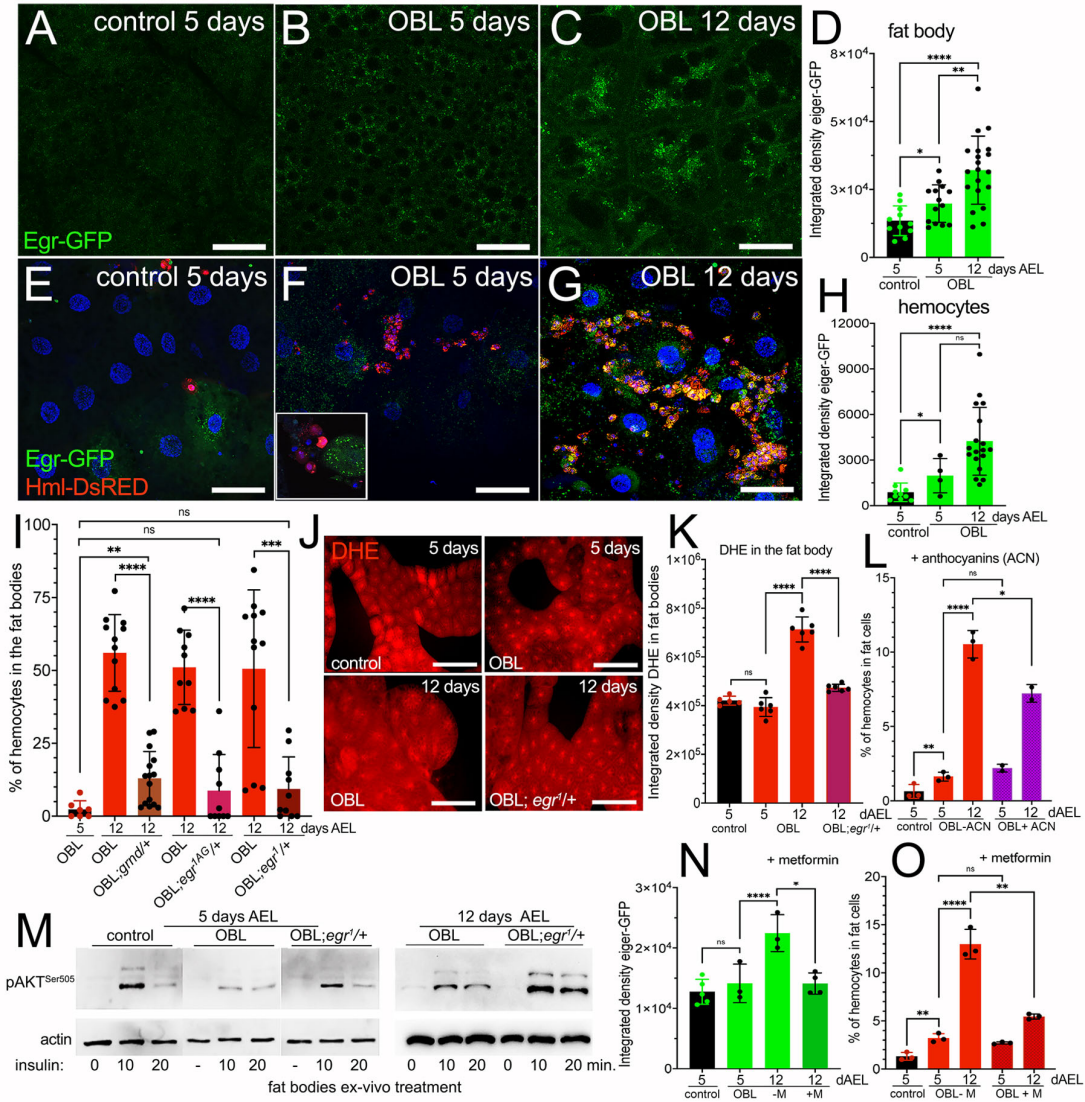
**Eiger signaling in OBL animals contributes to the infiltration of hemocytes, ROS production and insulin resistance.** (A-C) Confocal images showing Eiger-GFP expression in the FB from animals of the indicated age and genotype. Scale bars: 50 µm. (D) Quantification of Eiger-GFP expression was measured as integrated density in the FB of animals at the indicated time and genotype. (E-G) Confocal images of FB showing the presence of hemocytes expressing Hml-DsRed, and of Eiger-GFP. The inset in F highlights hemocytes (red) near FB cells expressing many vesicles containing Eiger-GFP. In G, aggregates of hemocytes (red) co-expressing Eiger-GFP are surrounding cells of the FB from OBL animals at 12 days AEL. Scale bars: 50 µm. (H) Quantification of Eiger-GFP measured in the hemocytes migrating in the FB; GFP is quantified as integrated density in FBs of animals at the indicated time and genotype. (I) Quantification of the number of hemocytes infiltrating the FB from OBL animals at 5 and 12 days AEL or in OBL animals heterozygous for *egr*^1^, *egr^1AG^* or *grnd* at 12 days AEL. (J) Dihydroethidium (DHE) staining in FBs. Scale bars: 50 µm. (K) Quantification of DHE staining measured as integrated density in the FBs from animals of the indicated days and genotype. (L) Quantification of the number of hemocytes infiltrating the FB in animals treated with anthocyanins (ACN). (M) Western blot using lysates from dissected FBs from animals of the indicated genotypes showing the level of Akt Ser505 phosphorylation upon treatment with insulin for the indicated time. Actin was used as a control for loading; western blot analysis was repeated twice using 20 FBs for each time point. (N,O) Quantification of Eiger-GFP expression (N) and the number of hemocytes in the FB (O), without metformin (−M) and after feeding the larvae 1 mM metformin (+M). *P*-values were calculated using unpaired two-tailed Student's *t*-test from *n*=10 larvae for each time point and genotype. Error bars indicate s.d. ns, not significant; *P<0.05, **P<0.01, ***P<0.001 and ****P<0.0001.

Inflammatory cytokines, including TNFα, play a dominant role in promoting chronic low-grade inflammation in adipose tissue (Ferrante, 2007). Also, in *Drosophila*, Eiger, the sole homolog of TNFα, controls fundamental cellular processes, including cell death and immune responses (Igaki and Miura, 2014). Using Eiger-GFP as a reporter to detect the level of Eiger by measuring the GFP (Destefanis et al., 2022; Sarov et al., 2016), we showed that, in the FB of control animals at 5 days AEL, Eiger was nearly undetectable. Conversely, it increased in the same-age OBL animals (Fig. 4A-D). Remarkably, at 12 days AEL, Eiger-GFP became visible in the FB of OBL larvae in numerous small cytoplasmic vesicles (Fig. 4C). The increase in Eiger protein was also accompanied by upregulation of *eiger* mRNA (Fig. S6). We then quantified the expression of Eiger-GFP in the hemocytes infiltrating the FBs by analyzing the level of GFP within the cells expressing Hml-DsRed. These data show that Eiger expression increased over time and was highest in hemocytes of OBL larvae at 12 days AEL (Fig. 4E-H). We also observed that hemocytes were more abundant in proximity to FB cells containing an elevated quantity of Eiger-GFP vesicles (Fig. 4F, inset), suggesting that non-autonomous signals may be released from the FB to attract the immune cells to eventually engulf and clear the dysfunctional adipose cells.

We then tested whether the migration of hemocytes into the FB depended on functional Eiger signaling, using mutants of e*iger* [*egr^1^*, *egr^3^* (Igaki et al., 2002) or *egr^1AG^*, *egr^3AG^* (Kodra et al., 2020)] and of the transmembrane receptor *grindelwald* (*grnd*), homolog of tumor necrosis factor receptor (TNFR) (Andersen et al., 2015). In these experiments, we observed a dominant effect of *eiger* mutations in reducing hemocyte infiltration in the OBL larvae. This analysis showed that OBL larvae carrying *grnd/+*, *eiger^1^*/+, and *egr^1AG^*/+ or *egr^3^*/+ in heterozygosity had significantly reduced numbers of hemocytes infiltrating the FB (Fig. 4I). Because similar results were found in animals carrying *egr^1AG^*/+ and *egr^1^*/+ (Fig. 4I), we continued our analysis using the *egr^1^* allele.

We previously showed that OBL animals had increased ROS levels in their FBs (Valenza et al., 2018). Thus, we analyzed whether ROS production in the FB of OBL larvae was dominantly dependent on Eiger signaling. These data showed that OBL animals heterozygous for *egr^1^* at 12 days AEL have significant inhibition of dihydroethidium (DHE) staining as an indication of reduced ROS levels (Fig. 4J,K).

We then verified whether Eiger expression was involved in the mechanism driving insulin resistance in FBs of the OBL animals. Using FBs of OBL or OBL; *egr^1^*/+ animals treated with insulin we found that Akt phosphorylation at Ser505 was significantly recovered in FB from OBL larvae heterozygous for *egr^1^*, suggesting that Eiger signaling could control the response to insulin in these cells (Fig. 4M).

In support of this hypothesis, we found that feeding OBL larvae the anti-diabetic drug metformin inhibits the upregulation of Eiger (measured by the expression of Eiger-GFP) (Fig. 4N) and inhibits hemocyte infiltration in the FB, which was significant in OBL animals at 12 days AEL (Fig. 4O). Likewise, oral administration of ACN significantly reduced FB hemocyte infiltration in the OBL larvae (Fig. 4L), reinforcing the role of ROS in this mechanism and corroborating our previous observation that feeding OBL animals ACN reduces the JNK/p46 phosphorylation signaling pathway that is often induced in response to oxidative stress and Eiger, which we correlated with ROS production in the FB of OBL animals (Valenza et al., 2018).

Overall, these data indicate that the mechanisms controlling insulin resistance and linked to oxidative stress are conserved in the fat cells of our OBL model.

### Imd and Toll pathways contribute to the mechanism of hemocyte infiltration in the FB of OBL larvae

**Fig. 5.**
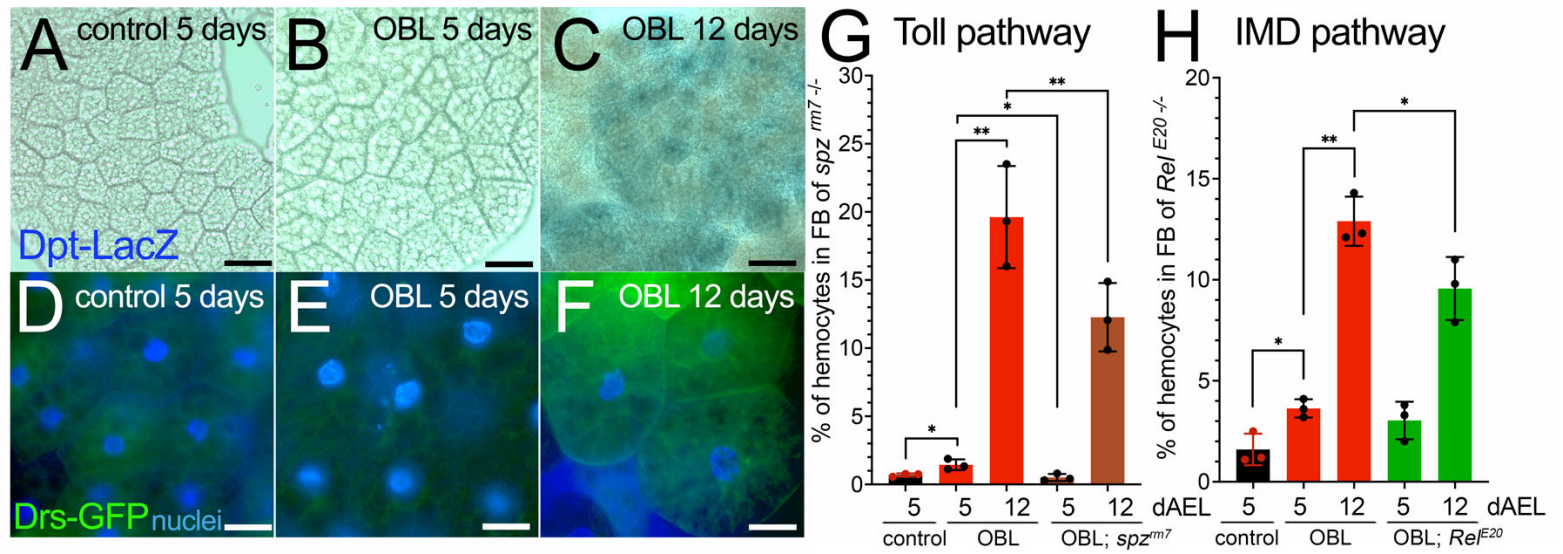
**Spaetzle and Relish are involved in the mechanisms controlling hemocyte infiltration in the FB in OBL animals.** (A-C) Confocal images of FB, showing activation of the reporter for Diptericin (*Dpt-lacZ*) at 12 days AEL in the FB of OBL animals. Scale bars: 20 µm. (D-F) Confocal images of FB showing the expression of GFP-positive cells representing activation of the reporter for Drosomycin using the *Drs-GFP* reporter line in control and OBL animals at the indicated time of development. Nuclei are stained with Hoechst. Scale bars: 50 µm. (G,H) Graphs indicating the percentage of hemocytes in the FB from the indicated genotypes. Dots indicate the independent experiments in which at least ten animals were used for each time and genotype. Statistical analysis was performed using unpaired two-tailed Student's *t*-test. Error bars indicate s.d. *P<0.05, **P<0.01.

Another mechanism that activates hemocytes in defense against infections and bacteria is driven by the Imd and Toll pathways in which Spaetzle (Spz), the ligand of the Toll receptor, initiates a humoral response in the FB that causes the secretion of antimicrobial peptides, including Diptericin (Dpt) and Drosomycin (Drs) (Lemaitre and Hoffmann, 2007). Interestingly, we found activation of the reporters Dpt-LacZ and Drs-GFP in the majority of the cells of the FB in OBL animals at 12 days AEL (Fig. 5A-E). Additional experiments using the mutant *spaetzle* (*spzr^m7^*) allele (Morisato and Anderson, 1994) showed that the number of hemocytes was significantly decreased in the FB of *spzr^m7^* mutants (Fig. 5G). Similar results were obtained using the *Rel^E20^* allele (Fig. 5D-F,H), which carries a mutation for the gene *Relish* (Hedengren et al., 1999), the homolog of human NF-κB that in flies acts downstream of both the Imd and Toll pathways (Lemaitre and Hoffmann, 2007).

These data suggest that cellular and humoral immunity cooperate to activate defense mechanisms, either cell mediated or with the induction of secreted factors induced by the cells when lipid signaling is dysregulated in the FB to attract hemocytes and eliminate them.

## DISCUSSION

### Hormonal regulation

Steroid hormones, like ecdysone in flies and estrogen/testosterone in humans, control the timing and progression of an animal's maturation. These hormones are synthesized in specific glands (prothoracic gland in flies and gonads in mammals) and released into the bloodstream to act on target tissues. They bind to specific receptors and initiate signaling cascades that result in the onset of maturation processes. In *Drosophila*, ecdysone triggers the transition from larval to pupal and adult stages (Kannangara et al., 2021). Similarly, in humans, steroid hormones are essential for the onset of puberty and sexual maturation. In addition to their roles in growth and maturation, steroid hormones such as estrogen and testosterone in mammals have been implicated in metabolic regulation, including obesity.

By reducing the steroid hormone ecdysone, we created a new obese-fly model (OBL) that we propose can be used to investigate obesity-related pathways, including insulin resistance, altered fat metabolism, chronic inflammation, immune system dysfunction and cardiac defects.

### Lipid metabolism

In obesity, adipose tissue undergoes hypertrophy and becomes dysfunctional. In our model, OBL animals showed hypertrophic fat cells (Fig. 2I). This tissue dysfunction resulted from an imbalance in lipolysis and lipogenesis; indeed, in the OBL animals, we observed an increase in TGs and elevated levels of FFAs in the whole body (Fig. 2F,G). Furthermore, the OBL animals showed an upregulation of *FAS* at the transcriptional level, a condition that in humans is induced by high glucose levels (Wong and Sul, 2010), resulting in *de novo* lipogenesis (Smith and Kahn, 2016), a process also conserved in flies (Miao et al., 2022). Our lipidomic analysis confirmed this situation and also showed that OBL animals had a substantial increase in SFAs (C14:0 myristic and C16:0 palmitic acids) over total lipids and of the MUFAs (C16:1 palmitoleic and C18:1 oleic acids) (Fig. 2H), physiologically toxic FFAs responsible in humans for the development of inflammation (Plotz et al., 2017; Sarabhai et al., 2020; Silva Figueiredo et al., 2017; Wrzosek et al., 2022). In flies, glucose levels affect the expression and activity of enzymes involved in lipid biosynthesis, including FAS (Mattila and Hietakangas, 2017). OBL larvae are hyperglycemic and have a higher basal level of insulin activation, measured by the tGPH reporter in the FB (Fig. 3I). These events and the abnormal distribution of DILP2 in the IPCs (Fig. 3B) may represent situations responsible for the transcriptional upregulation of FAS in our animals. In humans, *de novo* synthesis of fatty acids, especially in the liver, has been associated with insulin resistance (Kojta et al., 2020). The reduced response to insulin stimulation, seen in the cells of the FB of the OBL animals (Fig. 3I), may recapitulate some of the mechanisms described for insulin resistance in humans.

Our study also highlights significant contributions of Brummer, the homolog of human ATGL (Patel et al., 2022), in the mechanism that drives macrophage infiltration in the fat tissue (ATM). Previous research in flies has demonstrated that mutations in the *brummer* gene or alterations in its expression affect lipid metabolism and fat storage (Gronke et al., 2005). Here, we show that mutations in Brummer favor the migration of hemocytes in the FB when animals are reared on an HFD (Fig. 1A), and that, in OBL animals, Brummer is transcriptionally downregulated (Fig. 2A), suggesting control by upstream signals. In humans, ATGL expression has been found to be significantly reduced in obesity, decreasing the ability to break down stored TGs into fatty acids and contributing to fat accumulation in adipose tissue. This reduced lipolysis has been associated with insulin resistance and metabolic complications (Jocken et al., 2007). Insulin, in normal physiological conditions, suppresses lipolysis, but, in insulin-resistant states, this suppression might not work efficiently, leading to elevated and dysfunctional fat breakdown and the release of FFAs into the circulation, which, in addition to reduced ATGL activity, contributes to metabolic disorders and may favor ATM. We speculate that, in our OBL larvae, dysregulated lipolysis resulting from dysfunctional lipase expression could be responsible for the increased ratio of SFAs to total lipids (Fig. S3), leading to an increase in the synthesis of toxic lipids, such as C16:0 palmitic acid, C18:1 oleic acid (Fig. 2H), which could work as chemo-attractants for hemocytes into the FB.

Furthermore, because of the systemic lipid imbalance present in the OBL animals, the excess FFAs released by adipose tissue may be taken up by other organs (dyslipidemia) (Matsuda and Shimomura, 2013). Indeed, we observed an accumulation of lipid droplets within the pericardial cells of the tubular heart (Fig. 2O), which could be responsible for the impaired contractility of the heart cells (Fig. 2M). This reduced cardiac output can be used to screen for genetic factors or chemical drugs to improve heart function and find therapies for cardiovascular complications in obesity or related diseases.

Notably, the regulation of ATGL in human obesity is complex and can vary depending on individual factors and the specific stage of obesity. Its activity is influenced by growth hormone and glucocorticoids (Patel et al., 2022). Although in some cases ATGL is upregulated, in most cases its activity is impaired or reduced owing to factors such as chronic inflammation or dysregulated hormonal signaling, which is more similar to the mechanism we found in our OBL model in which Brummer/ATGL is reduced. Interestingly, in *Drosophila* Brummer, activity and expression are controlled indirectly by the hormone ecdysone, which we reduced to generate our hyperphagic animals (Valenza et al., 2018). Thus, understanding the role of lipases in obesity is still evolving. It is unclear whether directed mutations or reductions in Brummer/ATGL-like lipases contribute significantly to obesity; however, variations in genes related to lipid metabolism, including lipases, largely contribute to differences in obesity sensitivity, and reducing Brummer activity in *Drosophila* may provide insights into the regulation of lipid metabolism in human obesity.

### Glucose regulation

Previous studies in flies have shown that genetic manipulations of insulin signaling components can disrupt metabolic homeostasis, leading to abnormal glucose and lipid metabolism in the FB (Baker and Thummel, 2007). Here, we have created a nutrient overload situation in which hyperphagia and excessive food intake induce metabolic dysregulation and impair insulin-like signaling, leading to altered energy metabolism and nutrient storage. These conditions result in systemic metabolic imbalances, including ‘hyperglycemia’ (Fig. 3A) and aberrant glucose metabolism with the accumulation of glycogen and glucose in the whole body (Fig. S4).

Impaired insulin homeostasis is common in obesity and metabolic disorders. It may lead to insulin resistance, a condition that contributes to the development of type 2 diabetes. Although the term ‘insulin resistance’ may not be applicable to *Drosophila* in the same sense as in mammals, here we have identified a condition in which the *Drosophila* FB exhibits similarities to the insulin resistance seen in adipose tissue or liver in mammals (Fig. 3I). Indeed, the reduced response to insulin stimulation in the fat of the OBL animals, analyzed by tGPH translocation (Fig. 3I), and the lack of DILP2 regulation in the IPC cells in fed and starved conditions (Fig. 3B) suggest that these animals experience insulin resistance, with a high level of DILPs circulating in their hemolymph. In humans, prolonged exposure to high insulin levels leads to the downregulation of the insulin receptor on target cells, reducing their sensitivity to insulin and impairing downstream phosphorylation events. Here, we demonstrate that the FBs of OBL animals exhibit impaired responses to insulin, as evidenced by the expression levels of its indirect target 4EBP (Fig. S5). Moreover, the FBs acquire significant resistance to insulin treatment over time, indicated by the reduction in Akt phosphorylation at Ser505, the functional residue corresponding to Ser473 in vertebrate AKT.

### The immune system and inflammation – potential roles of Eiger/TNFα, and of the Imd and Toll pathways

In humans, adipocytes and other immune cells produce the secreted pro-inflammatory adipokine TNFα (Eiger in flies). Its secretion increases in conditions of obesity and is often associated with the chronic low-grade inflammation commonly observed in obesity-related diseases. Here, we show that the pro-inflammatory cytokine Eiger increases and is released by fat cells of the OBL animals, suggesting that the mechanism described in humans is conserved in flies (Fig. 4A-C). At the same time, hemocytes from OBL larvae upregulate Eiger when in contact with cells of the FB, suggesting the presence of non-autonomous signals between these cells induced by their touch. Studies in flies have shown that Eiger is released by hemocytes upon stress conditions, resulting in the activation of the JNK signaling pathway and apoptosis (Fogarty et al., 2016; Igaki and Miura, 2014; Parisi et al., 2014). A similar mechanism has been described in human adipose tissue from obese people where pro-inflammatory cytokines, including TNFα, are produced to attract macrophages, resulting in chronic low-grade inflammation (Ferrante, 2007). Thus, we propose that the condition of oxidative stress present in the FB of the OBL larvae increases Eiger expression (Fig. 4D), attracting the hemocytes into the FB (Fig. 1D-H and Fig. 4G). These data are supported by the transcriptional upregulation of *Glutathione S transferase D1* (*GstD1*) (Fig. S6), which is upregulated as a defense mechanism to counteract oxidative stress in response to the JNK/p46 pathway (Khoshnood et al., 2016), in the FB of OBL animals (Valenza et al., 2018).

Furthermore, we demonstrated that OBL animals heterozygous for *egr^1^*, *egr^3^* and *grnd* show significant reduction of hemocytes and ROS production in their FBs (Fig. 4I,J) and improved response to insulin with increased Akt phosphorylation at Ser505 (Fig. 4M). Interestingly, these results indicate a dominant effect of *eiger* and *grnd* mutations on the pathways, leading to the migration of the hemocytes into the FB. OBL animals have activated JNK/p46 signaling (Valenza et al., 2018) and insulin resistance, phenotypes indicating stress conditions. Thus, we hypothesize that these stress-induced cellular dysfunctions in OBL FBs promote favorable conditions that can be dominantly suppressed in the *eiger/+* background.

Eiger and Grnd are expressed in hemocytes and FB at similar levels, making it challenging to predict their respective role in these cell types. Further experiments are required to understand their specific contributions in the FB and during hemocyte migration. A combination of two binary systems (Parker and Struhl, 2020; Yagi et al., 2010) would be essential for such experiments. One system could reduce ecdysone in the prothoracic gland (OBL animal), and the other specifically targets Eiger components [Eiger and Grnd RNA interference (RNAi)] in either hemocytes or the FB using tissue-specific promoters. Notably, our findings suggest that hemocytes in OBL animals may adapt their metabolism to their environment and inter-organ signaling cues, as previously observed in their response to the antibacterial defense (Bajgar et al., 2021), possibly contributing to eliminating non-functional fat cells. Indeed, we showed the contribution of the Imd and Toll pathways to hemocyte migration in the FB of OBL animals (Fig. 5G,H). The Imd pathway activates Relish (*Drosophila* NF-κB homolog) (Stoven et al., 2000). Toll is another essential immune signaling pathway crucial in the defense against fungi and Gram-positive bacteria. Both are crucial for innate immune responses, generating antimicrobial peptides in response to microbial infection and stress (Lemaitre and Hoffmann, 2007). The roles of the Imd and Toll pathways in hemocyte migration remain unclear. Nevertheless, it is probable that these pathways collaborate with Eiger to facilitate immune activation and release signaling molecules that attract hemocytes to the FB when animals are undergoing stress, like in our model of obesity-related diseases.

### Impact on human diseases

In humans, inflammatory cytokines and hormones play a role in metabolic disorders, making the study of obesity-related inflammation complex, thus we the availability of a simple and genetically modifiable animal model such as *Drosophila* could allow for a better understanding of the role of Eiger/TNFα and other conserved cytokines in these metabolic diseases. Several *Drosophila* models have been developed that exhibit features like obesity and metabolic disorders. These models can be induced through genetic manipulations, high-calorie diets, or alterations in signaling pathways involved in metabolism and energy homeostasis. Our OBL model is new and mimics the reduction of estrogen or testosterone, which disrupts body fat distribution and is associated with increased risk of obesity-related metabolic disorders. In humans, obesity can also disrupt the hormonal balance in the body, including regulating steroid hormones (Fan et al., 2019; Sebo and Rodeheffer, 2021; Wittert and Grossmann, 2022). The interplay between steroid hormones, obesity and metabolic regulation is intricate and under investigation. Using appropriate *Drosophila* models can enhance our comprehension of these complex mechanisms. Finally, our OBL model can be a valuable tool because it provides a platform to perform genetic screens or test the efficacy and safety of novel therapeutic interventions. As shown here, administering the anti-diabetes drug metformin or antioxidants ACN significantly reduced immune cell infiltration. The OBL model can assess the impact of new drugs on various metabolic parameters, including body weight, fat accumulation, glucose tolerance, insulin sensitivity, adipose tissue metabolism and lipid accumulation profiles. Drug screening in *Drosophila* allows the rapid and cost-effective assessment of drug efficacy, toxicity and potential side effects.

In summary, although the specific molecular mechanisms underlying insulin resistance and metabolic dysregulation are still being elucidated, the conservation of crucial metabolic pathways between *Drosophila* and mammals increases the likelihood of identifying compounds potentially relevant to human diseases. Studies using our OBL model could provide valuable insights into the pathways and potential factors contributing to metabolic dysfunction, including non-alcoholic fatty liver disease (NAFLD), a condition that displays phenotypes similar to those observed in the FB of animals. Indeed, studies on metabolic alterations in NAFLD show that NAFLD is both a consequence of insulin resistance and a cause of it in the liver (Stefan et al., 2023). Thus, it is essential to build solid models to elucidate the complex interactions between NAFLD, insulin resistance and cellular senescence, which are all interconnected in various metabolic disorders (Spinelli et al., 2023).

## MATERIALS AND METHODS

### Fly stocks and husbandry

Fly cultures and crosses were raised at 25°C, on a standard medium containing 9 g/l agar (ZN5 B and V), 75 g/l corn flour, 60 g/l white sugar, 30 g/l brewer’s yeast (Fisher Scientific), 50 g/l fresh yeast and 50 ml/l molasses (Naturitas), along with nipagin and propionic acid (Fisher Scientific). The lines used were as follows: *P0206-Gal4* and *UAS-NOC1-RNAi* (B25992) (Destefanis et al., 2022; Valenza et al., 2018); *HmlΔ-DsRed* (referred to as *Hml-DsRed*) (Makhijani et al., 2011); lines mutant for *egr^1^*, *egr^3^*, *grnd*, *spzr^m7^* or *Rel^E20^* and the reporter lines *Dpt-LacZ* and *Drs-GFP* (a gift from Bruno Lemaitre, EPFL, Lausanne, Switzerland); *egr^1AG^*, *egr^3AG^* and *eiger-GFP* (fTRG library, VDRC-318615) (a gift from Hugo Stocker, ETH Zurich, Zurich, Switzerland).

### Hemocyte quantification

To label *in vivo* plasmatocytes, which comprise more than 95% of the hemocyte population in a *Drosophila* larva, we used the transgene line *Hml-DsRed* (Makhijani et al., 2011). FBs from larvae at 5 and 12 days AEL were dissected in phosphate-buffered saline (PBS) pH 7.4 and fixed in 4% paraformaldehyde (PFA) for 30 min at room temperature. Hoechst 33258 (Sigma Aldrich) was added to stain nuclei at the final concentration of 1 μg/ml. After washing with PBS, FBs were mounted onto slides with Vectashield (Vector Laboratories). Images were acquired using Leica TCS-SP5 or TCS-SP8 confocal microscopes, and the numbers of hemocytes and fat cells were counted from pictures taken at the same magnification.

### Immunofluorescence in hemocytes and IPC cells

#### Hemocytes in FB

Dissected FBs from 20 larvae were fixed in 4% PFA (Electron Microscopy Science) in PBS for 40 min. After permeabilization with 0.3% Triton/PBS, tissues were washed in 0.04% Tween 20 in PBS, saturated with 1% bovine serum albumin (BSA) in PBS and incubated with anti-SPARC antibody (1:400; Martinek et al., 2002) overnight. Alexa Fluor 555-conjugated anti-rabbit secondary antibody (Invitrogen) was used at 1:1000. After washing with PBS-Tween 20, FBs were mounted on slides using Vectashield (Vector Laboratories).

#### IPCs and anti-DILP2 immunostaining

Larvae of the indicated genotype at 5 or 12 days AEL were either kept in normal fly food or starved for 24 h in Petri dishes containing filter paper moistened with PBS. Brains were dissected in PBS and fixed in 4% PFA in PBS for 30 min and permeabilized in PBS-0.3% Triton X-100 (PBT) for 30 min. Tissues were blocked in 5% BSA in PBS, and incubated with rat anti-DILP2 overnight at 4°C (Geminard et al., 2009) followed by Alexa Fluor 488-conjugated anti-rat secondary antibody (Invitrogen). Tissues were washed and mounted in Vectashield with DAPI (Vector Laboratories), and fluorescence images were acquired using a Leica TCS-SP8 confocal microscope. Quantification of the mean pixels corresponding to the fluorescence intensity in the IPCs was determined using ImageJ, using similar parameters as previously described (Geminard et al., 2009; Parisi et al., 2013). Data are expressed in arbitrary units and represent the average fluorescence intensity in the IPCs of DILP2. Relative standard deviations were calculated using four to six independent experiments, each including at least ten animals of each genotype and condition.

### RNA extraction and quantitative real-time PCR (qRT-PCR) analysis

Total RNA was extracted from eight whole larvae, or from ten dissected FBs using an RNeasy Mini Kit (Qiagen) according to the manufacturer's instructions. Extracted RNAs were quantified using an ultraviolet (UV) spectrophotometer, and RNA integrity was confirmed with ethidium bromide staining. Then, 1 μg total RNA from each genotype was reverse transcribed into cDNA using SuperScript IV MILO Master Mix (Invitrogen). The obtained cDNA was used as the template for qRT-PCR using qPCR Mastermix (Promega). mRNA expression levels were normalized to *Actin 5C* mRNA used as the internal control The relative level for each gene was calculated using the 2-▵▵Ct method (Hulf et al., 2005) and reported as arbitrary units. Three independent experiments were performed, and cDNAs were used in triplicate.

The following primers were used for qRT-PCR: *Actin5c*-F 5′-CAGATCATGTTCGAGACCTTCAAC-3′, *Actin5c*-R 5′-ACGACCGGAGGCGTACAG-3′ (Parisi et al., 2013); *E74b* (*Eip74EF*)-F 5′-GAATCCGTAGCCTCCGACTGT-3′, *E74b*-R 5′-AGGAGGGAGAGTGGTGGTGTT-3′ (Parisi et al., 2013); *brummer*-F 5′-ATATGGACCCCGTGTTTCAA-3′, *brummer*-R 5′-AGCTTGTCGTGCTCCGTTAT-3′; *FAS*-F 5′-GTTGGGAGCGTGGTCTGTAT-3′, *FAS*-R 5′-GGTTTAGGCCAGCGTCAATA-3′; *Perilipin*-F 5′-AGGAAGATAATGTGCCAGTTCCCG-3′, *Perilipin*-R 5′-GCTGCCACCAGACTGCTCCAC-3′; *eiger*-F 5′-AAAGGTGGATGGCCTCACG-3′, *eiger*-R 5′-TGCCGGTATGTGCATTGTTG-3′; *GstD1*-F 5′-GACTCCCTGTACCCTAAGTGC-3′, *GstD1*-R 5′-TCGGCTACGGTAAGGGAGTCA-3′; *foxo*-F 5′-GCAATGTCGAGGAGCTGC-3′, *foxo*-R 5′-GCCCACTGCTGCTGTTGA-3′; *4EBP*-F 5′-ACAGCCAACGGTGAAACAC-3′, *4EBP*-R 5′-GCGTTGTTTGTATTTCGTTGC-3′.

### Larval weight

Larvae of the indicated age and genotypes were weighed as a group of 20 for each genotype and age for five independent experiments.

### Glucose, glycogen and TAGs, and measurement

Ten whole larvae were sonicated for 5 s on ice in 200 µl PBS for glucose assays, or in 0.1% PBS-Tween 20 for TG assays, heat inactivated at 70°C for 5 min to inactivate endogenous enzymes, before centrifugation at 4°C for 1 min at 1.9 ***g***. Supernatant was collected and centrifuged again at 4°C for 3 min at maximum speed and then used for the assays. Dissected FBs (10-20 for each genotype and age) were placed in an Eppendorf with 100 µl ice-cold PBS and sonicated following the same protocol as for whole larvae.

For glucose assays, trehalose was converted to glucose by adding Trehalase (Sigma-Aldrich, T8778) at a final concentration of 0.025 U/ml and incubating for 15 min at 37°C. The samples were mixed with 500 µl Glucose Reagent HK (Sigma-Aldrich, G3293) for 15 min at room temperature, and absorbance was measured at 340 nm according to the manufacturer's protocol. Concentrations were determined by comparing absorbances to a standard curve generated with a glucose standard (Sigma-Aldrich, G3285). Glucose measurements from undigested samples were subtracted to give the amount of glucose obtained from trehalose.

For hemolymph assays, hemolymph was pooled from ten larvae, and 1-2 µl was collected, diluted 1:20 in PBS and heat inactivated at 70°C for 5 min. For each glucose assay, 2 µl diluted hemolymph was used.

Glycogen was measured by digesting each sample with 0.3 U amyloglycosidase (Sigma-Aldrich, A7420) for 3 h at 37°C to convert glycogen to glucose. The samples were mixed with 500 µl Glucose Reagent HK (Sigma-Aldrich) for 15 min at room temperature, and absorbance was measured at 340 nm according to the manufacturer's protocol. Glucose measurements from undigested samples were subtracted to find the amount of glucose obtained from glycogen. A glucose standard from Sigma-Aldrich (G3285) was used to calculate sample concentrations.

TG measurements were performed by incubating 20 µl sample with 400 µl Free Glycerol Reagent (Sigma-Aldrich) for 15 min at RT, followed by 100 µl Triglyceride Reagent (Sigma-Aldrich) for 15 min, and absorbance was read at 540 nm according to the manufacturer's protocol. Concentrations were calculated by comparing absorbances to a standard curve generated using a TG standard contained in the kit. Glucose and TG concentrations were normalized against the total protein concentration in each sample as measured by bicinchoninic acid (BCA) assay (Pierce). Assays were repeated a minimum of three times.

### Lipidomic analysis

FBs from *Drosophila* were mechanically homogenized (IKA T25 Ultra-Turrax) in glass vials with 2:1 chloroform/methanol plus 0.01% butylated hydroxytoluene (BHT) as antioxidant. Lipids were extracted after the addition of 0.05% KCl plus a known amount of triheptadecanoin as an internal standard. Vials were shaken for 2 h at 4°C, and the organic phase was extracted after centrifugation at 0.5 ***g*** for 15 min. Later, a second extraction was performed, and the organic phases were merged, dried in a stream of nitrogen, resuspended in 2:1 chloroform/methanol plus BHT and stored at −80°C in the dark until use. The aqueous phase was dried, the resulting pellet was dissolved with 0.1 N NaOH, and the total protein amount was quantified utilizing BCA assay. All results were normalized to protein content.

### Qualitative and quantitative analysis of FB TGs

An aliquot of the lipid extract was loaded onto a channeled Silica TLC plate (Sigma-Aldrich), with a pre-run in 80:20:1 (v/v/v) hexane/diethyl ether/acetic acid, heat activated and developed up to 2.5 cm from the top. After the run, the TLC plates were sprayed with dichlorofluorescein (0.15% in ethanol), and, after drying in a stream of nitrogen, the silica spots corresponding to those of the known standards were scraped off.

For TG determination, samples were derivatized with methanol/3N HCl for 20 min at 80°C and extracted with hexane/water prior gas-liquid chromatographic (GLC) analysis conducted on a DANI 1000 machine (DANI Instruments, Milan, Italy) equipped with a flame ionization detector, an autosampler and a 30 m, 0.32 mm, 0.25 µm MEGA-1 (Mega Columns, Legnano, Italy) fused silica column. GLC parameters were as follows: flow of hydrogen at a constant pressure of 1 bar; injector temperature from 150°C to 300°C; detector temperature of 350°C; oven temperature ranging from 150°C to 330°C, with appropriate gradients. The total run lasted 25 min. Chromatograms were recorded, and the area of each fatty acid peak was quantified by the dedicated Clarity Software (Clarity, Prague, Czech Republic). The mass of a TG was assessed by integrating the areas of the peaks of each fatty acid composing the lipid, followed by summing and comparing it with the internal standard. Data were expressed as relative % (qualitative analysis of fatty acids) or as μg total fatty acid/mg total protein.

### Cell size and Nile Red staining

FBs were dissected from larvae at the indicated days AEL, fixed for 30 min in 4% PFA in PBS, permeabilized for 10 min in 0.2% Triton X-100 in PBS and incubated for 30 min in 0.01 mM Nile Red Solution (Sigma-Aldrich), with 1:100 Alexa Fluor 488 Phalloidin to visualize the cytoskeleton through binding between Phalloidin and F-actin, and with Hoechst 33258 to stain nuclei. After washing, the FBs were mounted in Vectashield with DAPI (Vector Laboratories), fluorescence images were acquired using a Leica TCS-SP5 confocal microscope, and the area of adipose cells for each FB was calculated with ImageJ software.

### Measurement of cardiomyocyte contraction

Larvae expressing the Hand-GFP reporter (Lo et al., 2007), which marks cardiomyocytes and pericardial cells, of the indicated age and genotypes, were embedded in a commercially available mounting putty (UHU) to block their movement with the cardiac tube visible in their dorsal part (Fink et al., 2009). Movies of Hand-GFP contraction were taken, and data were elaborated using the Open-Source Physics (OPS) Software Tracker, Video Analysis and Modeling Tools (https://physlets.org/tracker/). At least 11-12 larvae for each genotype and age were used. Data were elaborated by measuring the number of contractions over time, and *P*-values were calculated using unpaired two-tailed Student's *t*-test.

### Analysis of tGPH localization

Activation of insulin signaling was analyzed using the reporter line tGPH carrying the PH domain fused with GFP that translocates in the plasma membrane as a sensor of PI3K activation (Britton et al., 2002). FBs were quickly dissected in artificial hemolymph (Fink et al., 2009) containing 108 mM NaCl, 5 mM KCl, 2 mM CaCl_2_, 8 mM MgCl_2_, 1 mM NaH_2_PO_4_, 4 mM NaHCO_3_, 15 mM HEPES, 10 mM sucrose and 5 mM trehalose, at pH 7.1, and placed in a 24-well plate at ∼20 fats for each time point. A 1 mM final concentration of insulin (porcine from Sigma-Aldrich) was added for the indicated time. After washing once with cold PBS, the FBs were fixed in 4% PFA and Hoechst 33258 (Sigma-Aldrich) and mounted on microscope slides using Vectashield (Vector Laboratories). Images were acquired with a confocal microscope (Leica TCS-SP5), and the localization of tGPH was analyzed in the cytoplasm or/and membrane from images of ∼25-30 cells and quantified using ImageJ.

### Protein extractions and western blotting

The larval FBs (ten for each genotype) were dissected in Schneider's serum-free medium (Thermo Fisher Scientific) and lysed in 100 μl lysis buffer [50 mM HEPES/pH 7.4, 250 mM NaCl, 1 mM ethylenediaminetetraacetic acid (EDTA), 1.5% Triton X-100] containing a cocktail of phosphatase inhibitors (PhosSTOP 04906837001, Merck Life Science) and protease inhibitors (Roche cOmplete, Merck Life Science). Samples were sonicated three times for 10 s using an Ultrasonic Sonifier 250 (Branson, Darbury, CA, USA) equipped with a microtip set at 25% power. Tissue and cell debris were removed by centrifugation at 10,000 ***g*** for 30 min at 4°C. Proteins in the crude extract were quantified by a BCA Protein Assay Reagent Kit (Pierce), following the manufacturer's instructions with BSA as the standard protein. For SDS-PAGE, samples were incubated for 8 min at 100°C in standard reducing 1× loading buffer; 40 µg total protein was run on an SDS-polyacrylamide gel and transferred to nitrocellulose membranes (GE-Healthcare, Fisher Scientific, Italy). After blocking in 5% (w/v) non-fat milk in tris-buffered saline (TBS)-0.05% Tween 20 (TBS-T), membranes were incubated overnight with primary antibodies against *Drosophila* pAkt-Ser505 (1:500; Cell Signaling Technology, 4054), or Actin 5c (1:200; Developmental Studies Hybridoma Bank, JL20). Appropriate secondary antibody was incubated for 2 h at room temperature, followed by washing. The signal was revealed with a ChemiDoc Touch Imaging System (Bio-Rad).

### *In vivo* detection of ROS using DHE

DHE was used to detect cytosolic superoxides and ROS. The reaction between DHE and superoxide anions generates a highly specific red fluorescent product (ethidium), which intercalates with DNA. ROS levels were detected in FBs as described in Owusu-Ansah and Banerjee (2009). Briefly, larval FBs at 5 and 12 days AEL were dissected in Schneider's S2 medium (Gibco). After incubation in 30 μM DHE (Invitrogen) for 5-7 min in the dark and at room temperature, FBs were washed three times with Schneider's medium and immediately mounted with Vectashield Antifade Mounting Medium (Vector Laboratories). Images were taken using a Leica TCS-SP5 confocal microscope.

### Treatment with metformin, ACN and HFD

Crosses were kept in culture bottles perforated to provide adequate air circulation for the parents, and eggs were collected every 3 h on a grape agar plate supplemented with life yeast. After 24 h, first-instar larvae were placed into vials containing standard corn meal alone or supplemented with the different chemical drugs: 0.24 mg/ml ACN (gift from Katia Petroni and Chiara Tonelli, University of Milan) (Pilu et al., 2011), metformin (Abcam) at 1 mM final concentration, or HFD constituted of corn meal supplemented with 30% coconut oil (Trader Joe's, Pasadena, CA, USA).

### Statistical analysis

The experiments were repeated at least three times, and statistical analysis was performed by unpaired two-tailed Student's *t*-test or one-way ANOVA with Tukey's multiple comparisons, as indicated in the figure legends for each experiment, calculated using GraphPad Prism 7. *P*<0.05 was considered significant.

## Supplementary Material

10.1242/dmm.050388_sup1 Supplementary information
