# Supplementary material for: A Drosophila model targets Eiger/TNFα to alleviate obesity-related insulin resistance and macrophage infiltration
Source: Dis Model Mech. 2023 Nov 6;16(11):dmm050388. doi: 10.1242/dmm.050388 (PMC10651092; doi:10.1242/dmm.050388)
Supplement: Supplementary information [file dmm-16-050388-s1.pdf]

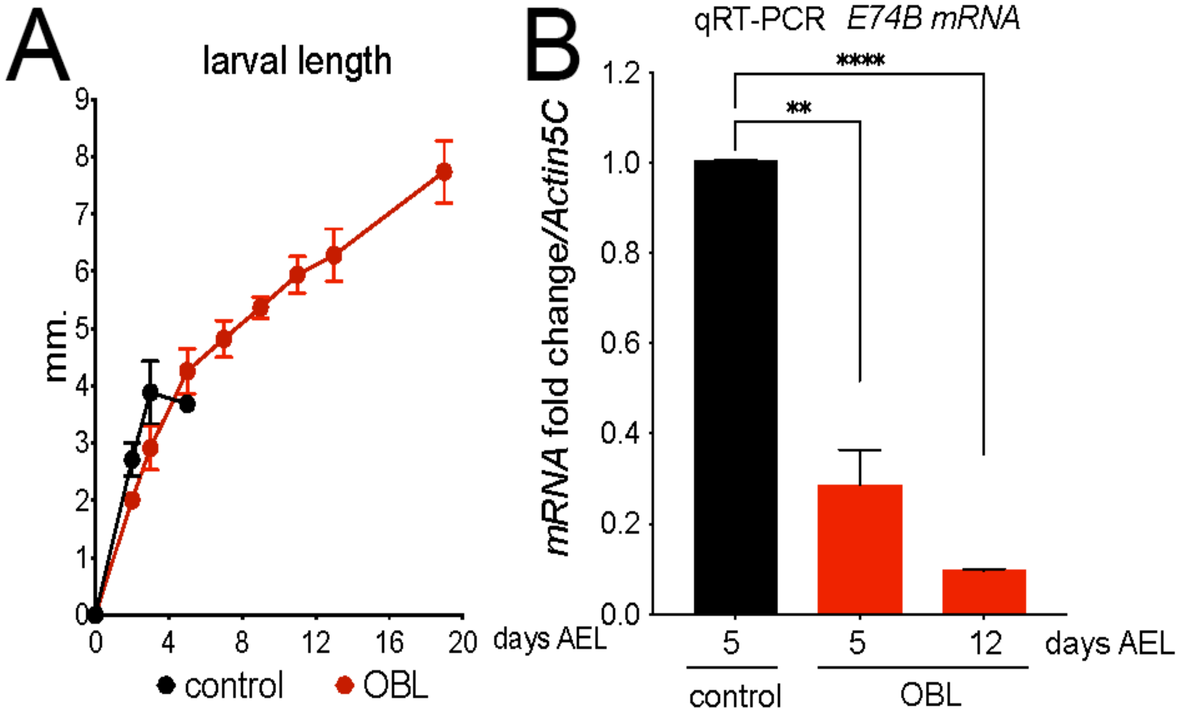


**Fig. S1. Larval growth and length and expression of *E74B-mRNA***

(A) Larval growth and length were measured over time of control *P0206>Gal4* or *P0206>Gal4; NOC1-RNAi* (OBL) larvae; *P0206>Gal4* larvae develop at the expected rate, and between 5-6 days AEL undergo to metamorphosis, while OBL animals keep growing. (B) expression of *E74B.* To develop the OBL model we reduced the level of ecdysone in the prothoracic gland (PG), by reducing the expression of the *NOC1* gene using the NOC1-RNAi transgene under the control of the *P0206-Gal4* promoter (Valenza et al., 2018). Reduction of NOC1 results in a strong reduction of the protein synthesis (Destefanis et al., 2022), which results in a decrease of Ecdysone production and circulation, shown by the decrease in the expression of its target E74B normally expressed in the FB. These animals, herein called OBL, *also show* a reduction of the size of their prothoracic gland (Destefanis et al., 2022), and because the level of ecdysone is low, they develop at a normal rate but never reach metamorphosis and continue to feed and gain weight until they reach an average of 20 days AEL before dying (Lin et al., 2011).

Disease Models & Mechanisms • Supplementary information

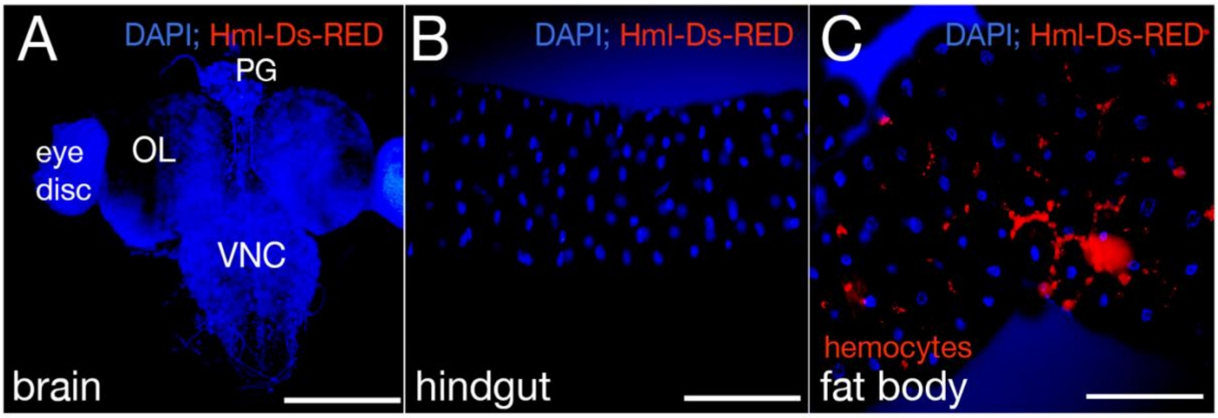


**Fig. S2. Hemocyte analysis in various tissues of OBL larvae at 12 days AEL.**
Confocal images of (A) brain, (B) hindgut, and (C) FB of larvae at 12 days AEL. Hemocytes are labeled in RED using the reporter line Hml-Ds-RFP (Makhijani et al., 2011), and the nuclei are stained with Hoechst in BLUE. VNC: Ventral Nerve Cord, OL: olfactory Lobe; PG, prothoracic gland. white bar represents 100 mm.

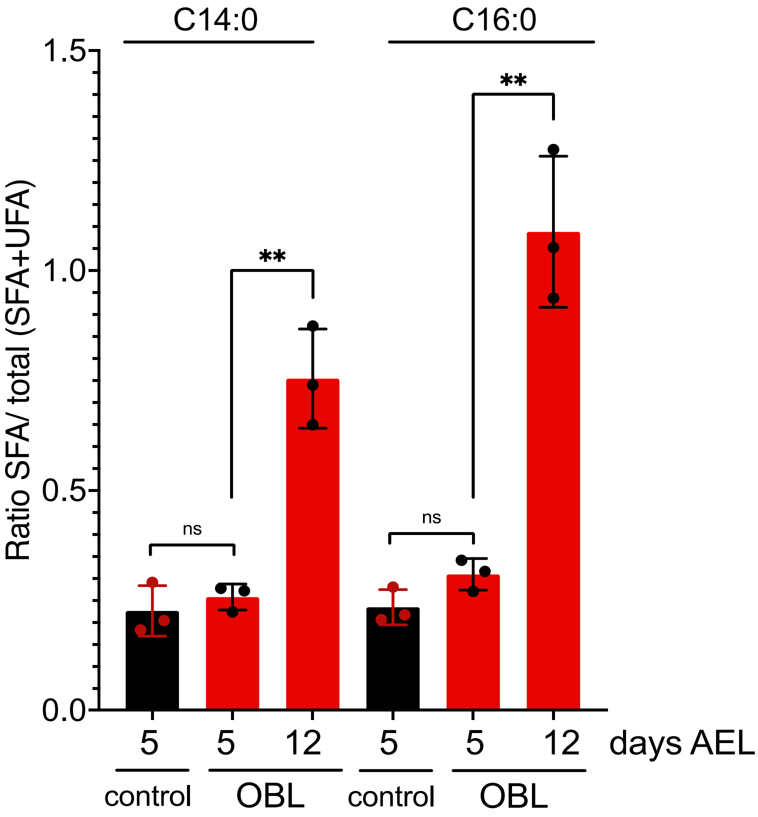


**Fig. S3. Lipidomic analysis showing the Ratio of C14:0 myristate and C16:0 palmitic Saturated Fatty Acids/Total lipids.**
Lipidomic analysis was performed in control and OBL larvae at the indicated days AEL. The graph represents the ratio of saturated fatty acids (SFA) over SFA plus unsaturated fatty acids (UFA). Statistical analysis was performed using one-way analysis of variance (ANOVA) with Tukey multiple comparisons. The asterisks represent the ** = $p < 0.01$, and the error bars indicate the standard deviations.

Disease Models & Mechanisms • Supplementary information

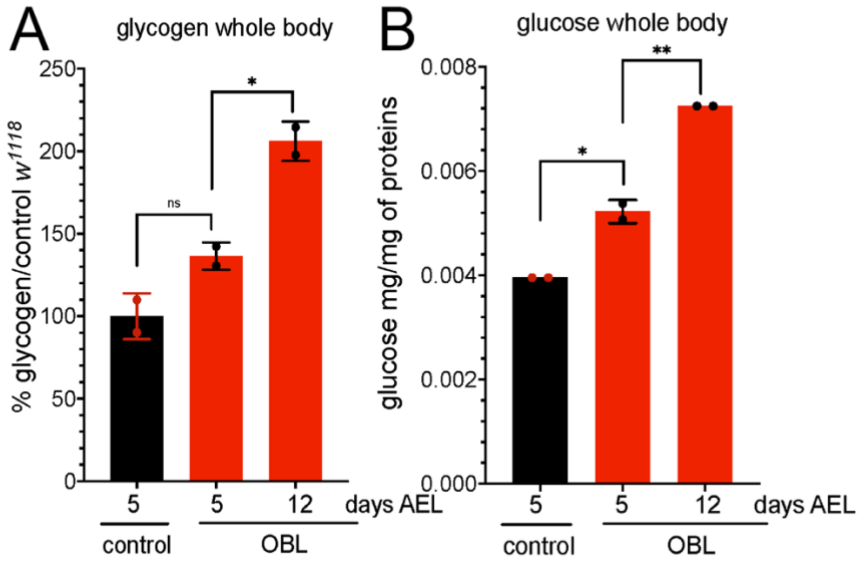


**Fig. S4. Quantification of Glycogen and Glucose in whole larvae**

Quantification of glycogen (A) or glucose from trehalose (B) was performed as described in material and methods using larvae of the indicated genotype and age. Statistical analysis was performed using Student's t-test. The asterisks represent the * = $p < 0.05$, ** = $p < 0.01$, and the error bars indicate the standard deviations.

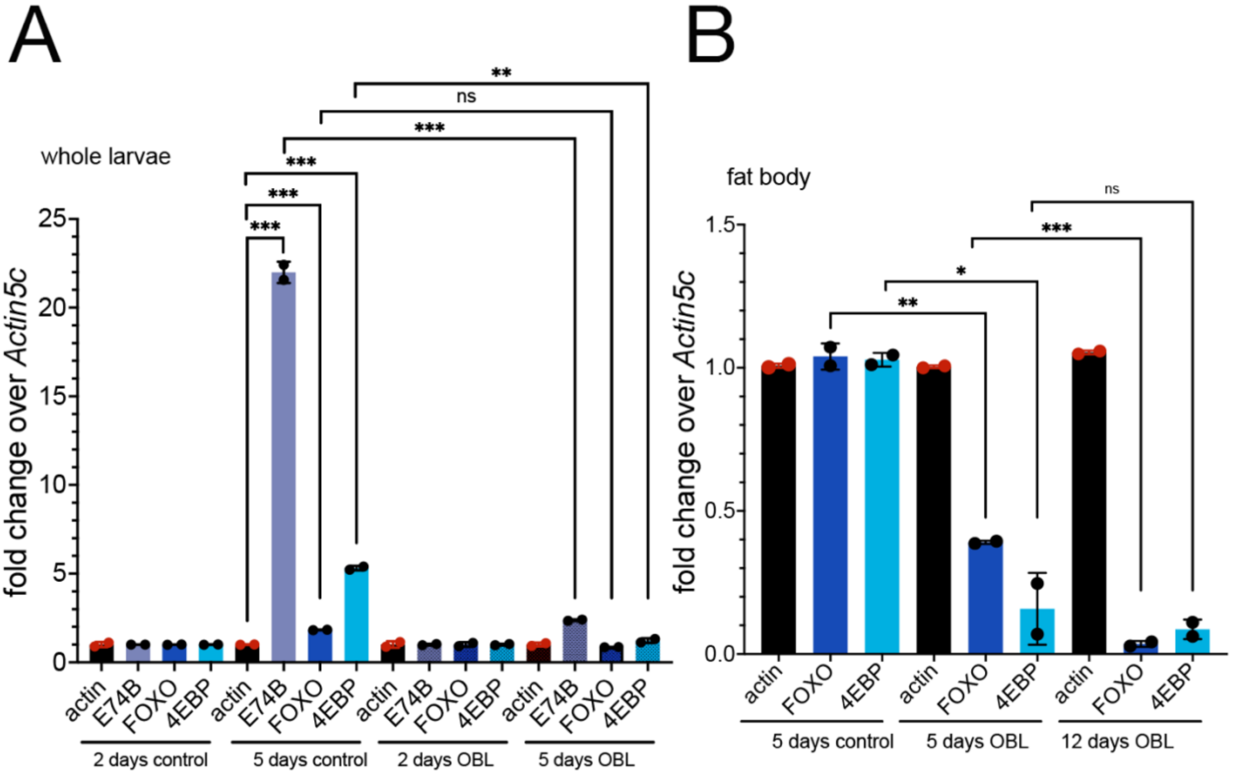


**Fig. S5. (A) *E74B, FOXO*, and *4EBP-mRNAs* expression in whole larvae from control or OBL larvae at 2 and 5 days AEL. (B) Expression of *FOXO,* and *4EBP-mRNAs* in the fat bodies from control and OBL larvae at 5 and 12 days AEL.**

qRT-PCT showing the level of *E74B, FOXO and 4EBP-mRNAs* from whole larvae (A) and FB (B) of the indicated genotype collected at 5 and 12 days AEL. Statistical analysis was performed using Student's t-test. The asterisks represent the * = $p < 0.05$, ** = $p < 0.01$, *** = $p < 0.001$, and the error bars indicate the standard deviations. At least 4 larvae and 20 fat bodies were used to collect RNA. Data are the average of two independent experiments.

Disease Models & Mechanisms • Supplementary information

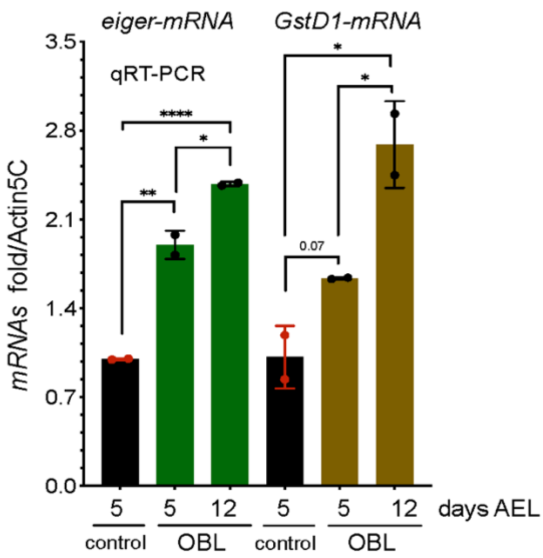


**Fig. S6. Expression of *eiger-mRNA* and *GstD1-mRNA* in control and OBL animals.** qRT-PCT showing the level of *eiger-mRNA* and of *GstD1-mRNAs* from whole larvae of the indicated genotype collected at 5 and 12 days AEL. Data are the average of two independent experiments.

## BIBLIOGRAPHY

**Destefanis, F., Manara, V., Santarelli, S., Zola, S., Brambilla, M., Viola, G., Maragno, P., Signoria, I., Viero, G., Pasini, M. E. et al.** (2022). Reduction of nucleolar NOC1 leads to the accumulation of pre-rRNAs and induces Xrp1, affecting growth and resulting in cell competition. *J Cell Sci* **135**.

**Lin, J. I., Mitchell, N. C., Kalcina, M., Tchoubrieva, E., Stewart, M. J., Marygold, S. J., Walker, C. D., Thomas, G., Leevers, S. J., Pearson, R. B. et al.** (2011). Drosophila ribosomal protein mutants control tissue growth non-autonomously via effects on the prothoracic gland and ecdysone. *PLoS Genet* **7**, e1002408.

**Makhijani, K., Alexander, B., Tanaka, T., Rulifson, E. and Bruckner, K.** (2011). The peripheral nervous system supports blood cell homing and survival in the Drosophila larva. *Development* **138**, 5379-91.

**Valenza, A., Bonfanti, C., Pasini, M. E. and Bellosta, P.** (2018). Anthocyanins Function as Anti-Inflammatory Agents in a Drosophila Model for Adipose Tissue Macrophage Infiltration. *Biomed Res Int* **2018**, 6413172.

Disease Models & Mechanisms • Supplementary information
